# Ear-ache

**Published:** 1892-09

**Authors:** 


### EAR-ACHE.

There is no more acute pain of childhood than ear-ache. This seems often to be caused by the sensitiveness to cold air of the tender membranes within the ear, and may be stopped by filling the ear with a little cotton dipped in sweet oil and warmed. If this does not give relief a few drops of laudanum, warmed by setting the bottle in hot water, may be added to the oil. A roasted onion is a favorite remedy with old women. If it is applied to the ear as hot as it can be borne it will relieve an obstinate case, and certainly is harmless.

When the pain is very intense it is better to dip the cotton, or, better still, a bit of wool, in hot laudamum alone, put it in the ear, and lay a hot bandage over it. It is a very bad practice to keep cotton in the ear any longer than is necessary, as such a habit will render the ear passages too sensitive and tender. When ear-ache appears in a grown person, and refuses to yield to simple remedies, a physician should be consulted at once, as a most serious disease may begin in this way. A "gathering in the head," as it is called in country parlance, is a painful and serious disease of childhood, as it may affect the hearing. It is very rare that the earwig or any other insect gets into the ear, but it is not an unknown thing, and when it occurs it causes an intense pain until the creature is smothered by pouring sweet oil into the ear. When cotton has been put into the ear and has served its purpose, it should be carefully removed and no bits left behind to work into the passages. Deafness is frequently caused by the presence of some such foreign body in the ear or by an accumulation of wax. In such a case the remedy consists in frequently syringing out the ear with warm water, using also a little sweet oil or white castile soap to dislodge the obstruction. Sometimes a large piece of wax comes out only after weeks of such syringing, and the defective hearing is suddenly restored.

